# Integrating BLUP, AMMI, and GGE Models to Explore GE Interactions for Adaptability and Stability of Winter Lentils (*Lens culinaris* Medik.)

**DOI:** 10.3390/plants12112079

**Published:** 2023-05-23

**Authors:** Md. Amir Hossain, Umakanta Sarker, Md. Golam Azam, Md. Shahriar Kobir, Rajib Roychowdhury, Sezai Ercisli, Daoud Ali, Shinya Oba, Kirill S. Golokhvast

**Affiliations:** 1 Department of Genetics and Plant Breeding, Faculty of Agriculture, Bangladesh Agricultural University, Mymensingh 2202, Bangladesh; 2 Department of Genetics and Plant Breeding, Faculty of Agriculture, Bangabandhu Sheikh Mujibur Rahman Agricultural University, Gazipur 1706, Bangladesh; 3 Pulses Research Centre, BARI, Ishurdi, Pabna 6620, Bangladesh; 4 Regional Agricultural Research Station, BARI, Jashore 7400, Bangladesh; 5 Department of Biotechnology, Visva-Bharati Central University, Santiniketan 731235, West Bengal, India; 6 Department of Horticulture, Faculty of Agriculture, Ataturk University, Erzurum 25240, Türkiye; 7 HGF Agro, Ata Teknokent, Erzurum 25240, Türkiye; 8 Department of Zoology, College of Science, King Saud University, P.O. Box 2455, Riyadh 11451, Saudi Arabia; 9 Laboratory of Field Science, Faculty of Applied Biological Sciences, Gifu University, Yanagido 1-1, Gifu 501-1193, Japan; 10 Siberian Federal Scientific Center of Agrobiotechnology RAS, 2b Centralnaya, Krasnoobsk 630501, Russia

**Keywords:** AMMI, BLUP, GGE biplot, regression and deviation from regression, non-parametric and parametric stability indices, lentil

## Abstract

Lentil yield is a complicated quantitative trait; it is significantly influenced by the environment. It is crucial for improving human health and nutritional security in the country as well as for a sustainable agricultural system. The study was laid out to determine the stable genotype through the collaboration of G × E by AMMI and GGE biplot and to identify the superior genotypes using 33 parametric and non-parametric stability statistics of 10 genotypes across four different conditions. The total G × E effect was divided into two primary components by the AMMI model. For days to flowering, days to maturity, plant height, pods per plant, and hundred seed weight, IPCA1 was significant and accounted for 83%, 75%, 100%, and 62%, respectively. Both IPCA1 and IPCA2 were non-significant for yield per plant and accounted for 62% of the overall G × E interaction. An estimated set of eight stability parameters showed strong positive correlations with mean seed yield, and these measurements can be utilized to choose stable genotypes. The productivity of lentils has varied greatly in the environment, ranging from 786 kg per ha in the MYM environment to 1658 kg per ha in the ISD environment, according to the AMMI biplot. Three genotypes (G8, G7, and G2) were shown to be the most stable based on non-parametric stability scores for grain yield. G8, G7, G2, and G5 were determined as the top lentil genotypes based on grain production using numerical stability metrics such as Francis’s coefficient of variation, Shukla stability value (σi^2^), and Wrick’s ecovalence (Wi). Genotypes G7, G10, and G4 were the most stable with the highest yield, according to BLUP-based simultaneous selection stability characteristics. The findings of graphic stability methods such as AMMI and GGE for identifying the high-yielding and stable lentil genotypes were very similar. While the GGE biplot indicated G2, G10, and G7 as the most stable and high-producing genotypes, AMMI analysis identified G2, G9, G10, and G7. These selected genotypes would be used to release a new variety. Considering all the stability models, such as Eberhart and Russell’s regression and deviation from regression, additive main effects, multiplicative interactions (AMMI) analysis, and GGE, the genotypes G2, G9, and G7 could be used as well-adapted genotypes with moderate grain yield in all tested environments.

## 1. Introduction

Lentil (*Lens culinaris* Medik.) is a nutrient-rich legume grown in many countries as a winter crop [1,2]. Lentils are also grown as summer crops in some countries of Europe and elsewhere. It has a comparatively large genome of 4063 Mpb and is a self-pollinated diploid legume (2n = 14 chromosomes) with a high annual value of cold season legumes crop [3]. In human nutrition, it provides a dependable supply of dietary proteins (22–25%), minerals (K, P, Fe, and Zn), carbohydrates, and vitamins [4]. They even offer additional health benefits due to their fat content and low glycemic index [5]. On a global scale, lentil ranks sixth in terms of output among the major legumes, after dry beans, peas, chickpeas, beans, and cowpeas, and it accounts for 6% of all dried legume production. Currently, it occupies 193,102 hectares of land in our nation, producing 251,805 tons and producing 1304 kg/ha, respectively [6]. Smallholder farmers in Bangladesh produce lentils because they are a valuable source of protein for rural households and also a source of money for the sale of seeds [7]. Farmers’ low-input practices and the use of certified seeds of improved varieties are crucial components that would considerably increase productivity and improve rural community’s livelihoods, flexibility, and food safety [8].

The low yield of lentil genotypes is caused by a combined effect of diverse production barriers, including poor soil fertility, a lack of high-yielding variations, the severity of moisture stress, diseases and insect pests, weeds, and inadequate crop management skills. Global climate change, particularly owing to precipitation and ecological change, has had a significant impact on lentil production. To maximize yield performance and control phenotypic expression, plant breeders must choose certain genotypes that are stable in or adaptable to a given environment. Stability means consistency of variety in an environmental fluctuation [9,10]. The environment plays a crucial role in genotypes’ environment interaction that influences crop production [11,12]. A complex character, yield is predominately dependent on many agronomic traits [13,14]. Many environmental and genetic factors significantly influenced it [15,16,17,18]. Numerous breeding initiatives aim to increase lentil yield performance. The complicated attribute of yield is influenced by many genes and is greatly influenced by the environment.

The degree of genotype–environment (G × E) interaction may be analyzed using various numerical and graphical stability methods, which can also be used to identify genotypes with high seed yields and stability under various environmental circumstances. There are two types of numerical methods for modeling the effects of G and E interactions. Parametric methods include Wricke’s [19] ecovalence (Wi^2^), the regression coefficient (bi), deviation from regression (S^2^di) parameters [20,21,22], stability variance (r^2^), Francis and Kannenberg’s [23] coefficient of variance, and AMMI-based stability parameters [24,25]. These stability statistics have favorable characteristics when interaction effects and the normal distribution of errors are taken into account statistically, but they could not be applicable if these assumptions are not working [26]. Based on the ranks of genotypes in each environment, non-parametric methods, such as Huehn’s [27] and Nassar and Huehn’s [28] non-parametric measures S^(i)^ and Thennarasu’s non-parametric [29] measures NP^(i)^, have been proposed to interpret and describe the responses of genotypes to various environments. In general, each parametric and non-parametric technique depicts the phenomena of G × E interaction in a particular way, and each method has advantages and disadvantages for choosing desirable genotypes. Therefore, to assess the stability and adaptability of genotypes, plant breeders employ both parametric and non-parametric approaches [30].

Numerous statistical techniques have been planned to take advantage of the positive G × E interaction and help the breeding program’s choices of cultivars and recommendations for the set of target environments [31]. However, the major additive effect and the multiplicative interaction model (AMMI) have often been utilized to explore the G × E interaction for different diseases in diverse crops [32,33]. Plant breeders need to comprehend the GEI pattern among test entries in multi-environment trials (METs). The phenotypic stability of the genotypes for several places or a specific genotype for different environmental circumstances may be determined with the help of modeling the G × E in METs [30,34]. There are other methods for analyzing genotype stability, including biplots derived from additive main effects and multiplicative interaction [35] and the genotype plus genotype–environment interaction [36,37]. However, the main restriction is that they may only be considered representative when two principal components (PCs) are important.

The AMMI Stability Index (ASI) and AMMI Stability Value (ASV) using two PCs, as well as the Modified AMMI Stability Index (MASI) and Modified AMMI Stability Value (MASV), using all the significant PCs, were investigated to more clearly show the stability of genotypes after the necessary modifications. For choosing a stable genotype with high yields, several selection indices were created such as selection index [38], parametric and non-parametric genotype selection index [39], and others. These indices direct the simultaneous choice of both stability and high yield using information from yield and stability parameters. It is possible to argue that selection techniques that use yield as the only criterion are preferable to those that combine stability and adaptability into a single statistic [40]. The relative performance of genetic values (RPVG), harmonic mean of the relative performance of genetic values (HMRPGC), and harmonic mean of genetic values (HMGV) predicted values are based on mixed models, which have been used to interpret the genotypic stability and adaptability of perennial plants as alternatives to the simulation of selection by yield, stability, and adaptability [41]. The GGE biplot analysis is an extremely helpful graphical tool since it offers visual pictures and a clear summary of the main data and outcomes [42]. The major effect of the genotype plus the GE interaction biplot (GGE biplot) is the best GGE biplot approach because it can visually demonstrate the general suitability of environments in terms of response to selection [43,44].

The GGE biplot (genotypes main effect plus G × E interaction), one of the graphic stability techniques, has already been used to represent G × E interaction in METs in several crops [45]. This estimate has been applied to several major crops, for example, mungbean (*Vigna radiata*) [46] and lentil [2]. Therefore, the goals of this study were to (1) analyze the GEI for grain yield using several stability methods, including parametric, non-parametric, BLUP, GGE biplot, and AMMI, (2) ascertain the relationships between the various stability methods, and (3) find the best genotypes of lentils based on high yield and stability.

## 2. Results

### 2.1. Stability Analysis for Different Yield Contributing Characters

Stability parameters regression coefficient (bi) and deviation from regression (S^2^di) were estimated for the studied traits following Eberhart and Russell’s model [20]. The regression coefficient ‘bi’ is used to identify stable genotypes [21,47]. A genotype with a bi value <1.0 has above-average stability and is specially adapted to low-performing environments. On the other hand, a cultivar with a bi value >1.0 has below-average stability and is specially adapted to high-performing environments, and a cultivar with a bi value equal to 1.0 is stable and well-adapted to all environments [21]. According to Eberhart and Russel [20], the genotype was expressed to be stable when it showed a regression coefficient of unity (bi = 1) and a minimum deviation from the regression (S^2^di = 0). In this model, the regression coefficient (bi) is considered a parameter of response, and deviation from regression (S^2^di) is the parameter of stability. The relatively lower value of bi (around 1) means less responsive to environmental change and therefore more adaptive. If bi is negative, the genotype may be grown only in a poor environment. If S^2^di is significantly different from zero, it invalidates the linear prediction. If S^2^di is non-significant, the performances of a genotype for a given environment may be predicted. Therefore, a genotype whose performance for a given environment can be predicted if S^2^di ~0 is a stable genotype. The environmental index (Ij) directly reflects the poor or rich environment in terms of negative and positive Ij, respectively.

A lower number of days to flowering ensures short duration and earliness, so a lower phenotypic index (Pi) for days to flowering had an evidentiary contribution to ensure the development of short-duration variety. Taken into the mean, Pi, bi, S^2^di, and enumerating the genotypic response and adaptability under different environmental conditions, the genotypes G10, G3, G2, and G8 assumed positive Pi, non-significant bi, and S^2^di point out stable performance for days to flowering to all the locations. Jashore and Mymensingh acquired the favorable location, and Ishwardi was the unfavorable location for days to flowering (Table 1).

For days to maturity, the genotype G2 displayed a lower Pi, significant bi, and S^2^di were near zero, which was stable for a favorable environment such as Rangpur. The genotypes G1, G5, G6, G9, and G10 displayed positive Pi, non-significant bi, and significant S^2^di, suggesting that the stability of these genotypic growth durations was unpredictable due to significant S^2^di. The genotypes G8 displayed positive Pi, non-significant bi, and non-significant S^2^di, suggesting stability across four locations. The environmental index (Ij) directly reflects the rich or poor environment in terms of negative and positive Ij, respectively, for days to maturity (Table 2). Consequently, when days to maturity were considered, the environmental index Ij was maximum in Ishwardi and minimum in Rangpur, indicating that Rangpur provided the most suitable environment for days to maturity.

The genotypes G3, G6, and G2 showed negative Pi for plant height, non-significant bi, and S2di, which indicated the stability of genotypes with shorter plant stature (Table 3). A shorter variety is required to maintain lodging resistance. Genotype G7 had positive Pi, significant bi, and non-significant S2di, which revealed that this genotype was suitable for a specific location at Ishwardi, Jashore, and Rangpur. G4 and G8 demonstrated positive Pi, negative and non-significant bi, and negative and non-significant S^2^di, noticing that genotypes were stable in all locations with semi-dwarf plant stature (Table 3).

For the pods per plant, the positive Pi represents the higher pods per plant, and the negative Pi represents the lower pods per plant among the genotypes. Again, positive and negative Ij reflects the rich or favorable and poor or unfavorable environments for this character (Table 4). Genotypes G8, G4, and G2 had positive Pi, non-significant bi, and non-significant S^2^di, informing that these were stable for pods per plant for all the locations. G6 exposed positive Pi, negative and non-significant bi as well as positive and significant S^2^di, which notifies that this genotype was reactive to the locations Jashore and Rangpur (Table 4). Considering the mean, bi, and S^2^di, it was evident that all the genotypes exhibit different responses of adaptability under different environmental conditions. Low mean and positive Pi with non-significant bi and non-significant S^2^di had been recorded in the genotypes G1 and G9.

A higher 100 seeds’ weight and positive Pi confirmed a higher yield. The bi values for 100 seeds’ weight ranged from 0.95 to 2.41; these differences in bi values notify that all genotypes responded differently to different environments. High and considerable positive Pi, non-significant bi, and S^2^di were recorded in the genotypes G8, G6, G7, and G1, which indicated the genotypes were stable over all the locations (Table 5). The genotype G10 displayed a positive Pi, significant bi, and S^2^di was zero (S^2^di ~0), indicating the 100 seeds’ weight was highly responsive to the location of Ishwardi, Jashore, and Rangpur.

A high mean and positive Pi for seed yield were some of our desired characteristics for measuring stability. The genotypes G6, G7, G9, and G10 exposed positive Pi, non-significant bi, and S^2^di, notifying that they were stable over all locations. G8 and G1 gained low mean and positive Pi, non-significant bi, and S^2^di, which suggested that these genotypes were stable over all the locations, but their stability was not considered because of the lower yield performance (Table 6). Jashore and Ishwardi were favorable locations, but Mymensingh and Rangpur were unfavorable locations for seed yield.

### 2.2. AMMI Analysis of Variance

In the current investigation, the AMMI ANOVA was introduced with regard to the yield performance of 10 potential lentil genotypes evaluated in four environments over the *rabi* seasons in Table 7. The AMMI model is frequently used in stability analysis because it recommends a preliminary diagnosis of the model to be fitted into multiple environmental evaluations, enables partitioning of the genotype—environment interaction, and explains patterns and relationships between genotypes and environments [35,48]. The findings of the AMMI study demonstrated that genotype, environment, and GEI had a substantial impact on DF, DM, PH, PPP, HSW, and seed yield (Table 7). According to the AMMI analysis of variance, the effect of genotype (G) accounted for 39.87% of the total sum of squares (SS) for DF, whereas the environmental (E) effects and GEI accounted for 24.43% and 29.90% of the total SS, respectively. According to DM, environmental factors accounted for 27.92% of the total sum of squares, while genotypic factors accounted for 40.62%, and GE effects accounted for 30.65 % (Table 7). The genotype-by-environment interaction accounted for the largest share (53.78%) of the sum of squares from the total treatment, which was followed by the genotype’s main effect (24.98%), and the effect of environment, which was 6.62%. For PPP, G, E, and GEI each account for 8.78%, 9.09%, and 81.47% of the total sum of squares. A total of 88.76%, 8.78%, and 2.51% of the HSW came from G, E, and GEI, respectively. The sum of squares for genotype main effects comprised 69.14% of the total variation in the analysis of variance, and this component had the greatest impact on seed yield. Environmental variations accounted for 22.79% of the overall variance in seed yield, while GE interaction effects accounted for 8.01% (Table 7). Three significant interaction main components were identified from the interaction-related multiplicative variance of the treatment sum of squares. Overall, 96.60% of the GEI sum of squares for DF, 95.40% for DM, 99.9% for PH, 99.98% for PPP, and 99.60% for HSW were explained by AMMI2 (IPCA1 + IPCA2) (Table 7). The first principal component (IPCAI) and second principal component (IPCA2) explained 61.30% and 23.30% of the total GE variance (84.60%), respectively, in the AMMI analysis for seed yield (Table 7). With huge variances between genotypes accounting for the majority of the variation in seed yield, a large sum of squares for genotypes revealed that the genotypes were varied, demonstrating that genotypes had a significant impact on seed yield [49]. A significant difference in yield that could be explained by genotypes suggested that the genotypes were unique and that changes in the genotype were mostly variations in yield. The sum of the squares for the environments was moderate, indicating that the environments investigated in this study were distinct and that there were also variations in the environmental means responsible for the variable genotypic response with regard to yield. This is frequently concurring with Akter et al. [50]. Previous findings confirmed that employing the first two PCAs may explain the greatest GEI in the majority of cases [51].

### 2.3. Additive Main Effects and Multiplicative Interaction 1 (AMMI1)

**Figure 1:**
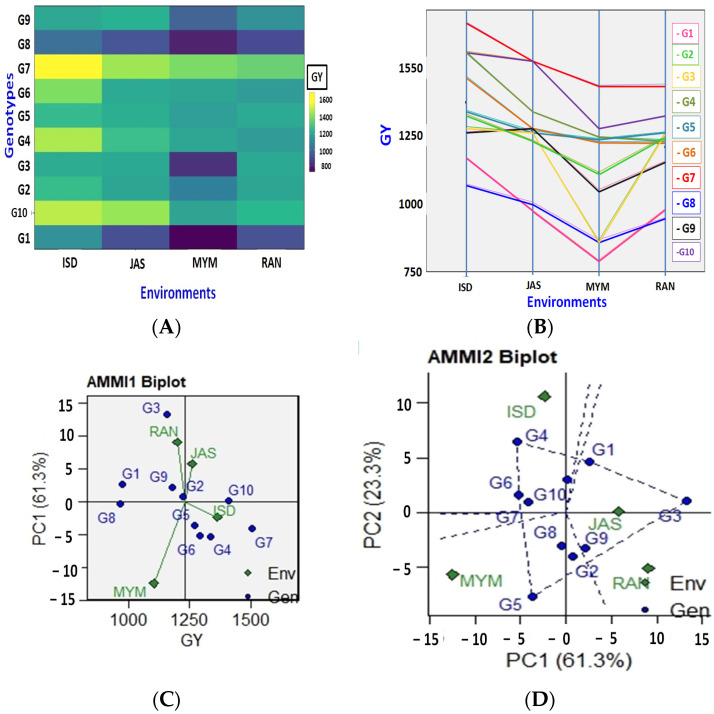
The seed yield variation of the studied ten lentil genotypes across four environments (**A**,**B**). The AMMI1 and AMMI2 biplot (yield vs. principal component 1 (PC1)) for grain yield (kg/ha) of 10 lentil genotypes evaluated under four environments ((**C**,**D**), respectively).

**Figure 2:**
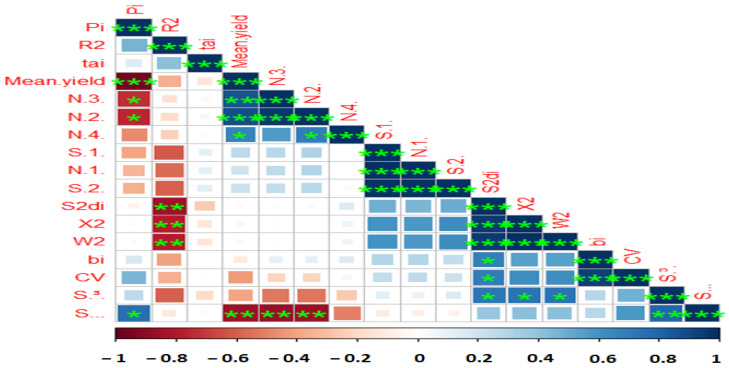
Correlation among various stability parameters with yield data of 10 lentil genotypes evaluated under four test environments. AMGE—sum across environments of genotype × environment interaction (GEI) modeled by AMMI; ASI, AMMI Stability Index; ASV, AMMI Stability Value; ASTAB, AMMI-based stability parameter; AVAMGE—sum across environments of the absolute value of GEI modeled by AMMI; DA—Annicchiarico’s D parameter; DZ—Zhang’s D parameter; EV—averages of the squared eigenvector values; FA stability measure based on fitted AMMI model; MASI—Modified AMMI Stability Index; MASV—Modified AMMI Stability Value; SIPC—sums of the absolute value of the IPC scores; Za—absolute value of the relative contribution of IPCs to the interaction. *, **, and *** Significant at 0.05, 0.01, and 0.001 probability levels, respectively.

This finding is supported by the difference between the mean yield of the tested genotypes, which ranged from 777 kg per ha (G1 in environment MYM) to 1665 kg per ha (G7 in environment ISD) (Figure 1A and Figure 2B). Moreover, Figure 1A demonstrates that environment production was highly changeable and varied from environment to environment. The G1 genotype was discovered to have the lowest yield at two sites (JAS and MYM), whereas the G8 genotype was detected at sites in ISD and RAN (Figure 1A and Figure 2B). In every site, genotype G7 had the highest yield (Figure 1B). The biplot abscissa and ordinate in AMMI1 show the main effect and first principal component (PC1) term, respectively. According to Murphy et al. [52], genotypes with identical PC1 signs and scores but higher PC1 scores are those that can adapt to a particular environment. The genotype with PC1 values that are closest to zero suggests general adaptability in all environments. When a gene and environment have a comparable sign on the PCA pivot, their interaction is positive; otherwise, it is negative. If a genotype or environment has a PCA1 score that is near zero, it has minimal interaction impact. Figure 1C shows the distribution of test conditions and genotypes of lentils based on mean grain yield and IPC1 scores. The AMMI1 biplot for the grain yield of the four environments and ten lentil genotypes using genotypic and environmental scores is shown in Figure 1C. According to this graph, the genotypes or environments to the right of the perpendicular lines have higher average values that are greater than those to the left side. In this biplot, a line perpendicular to the horizontal represents the mean total grain yield (1232 kg per hectare). G7 had the highest mean grain yield, which was followed by G10, G4, G6, and G5, with respective yields of 1507, 1411, 1336, and 1292 kg per hectare. Half of the genotypes had greater mean grain yields than the total. The lowest mean grain production was seen in genotypes G8 (967 kg per ha), G1 (973 kg per ha), and G3 (1159 kg per ha) (Figure 1C). The results showed that the G10 genotype was very productive and stable, whereas G7 and G4 were similarly productive and a little stable. G8 is a low-yielding genotype that ought to be stable. Based on yield performance, the MYM environment was poor, but the ISD and JAS environments were rich. The initial PCA1 results for genotypes G10, G7, and G5 were nearly zero, indicating that these genotypes were less impacted by their surroundings (stable genotypes). Out of them, G10, G5, and the almost zero IPCA1 scores were evaluated as the genotypes with the highest yields and most stable across all or most environments.

### 2.4. Additive Main Effects and Multiplicative Interaction 2 (AMMI2)

The PC1 and PC2 were utilized in AMMI2 for the abscissa biplot and ordinate, respectively. According to this biplot (Figure 1D), environments and genotypes close to the biplot’s origin have the least impact on GEI, whereas habitats and genotypes that are far from the biplot’s origin have the most influence on GEI. As a result, in comparison to other genotypes, those closest to the origin were thought to be stable. Highly interacting genotypes were those genotypes that broke down from the beginning. The genotypes G7, G6, and G10 expressed either favorably or negatively high interactive behaviors in the current study, or they contributed more to the observed GE interaction. When compared to the JAS and RAN environments, the G3 genotype performed the best. G4 and G5 genotypes were adapted to ISD and MYM, respectively. Since G7 and G10 are less affected by the G × E interaction, they would perform well in various environments. Through lateral lines, the environmental scores are linked to their point of origin. Short arrow sites do not impose a lot of interaction forces. Long arrows engage in the strong interaction. The genotypes near the ordinates reflected a more adaptation to the surroundings, whilst the following genotypes expressed a more specific adaptation to the environment [20]. The nature of G × E is governed by the angle between the genotype and the environmental vectors: it is positive for acute angles, insignificant for straight angles, and negative for obtuse angles. The magnitude of G × E demonstrated by genotypes over the environment, or environments over genotype was also indicated by the space of genotype and environment vectors from the biplot origin.

### 2.5. Non-Parametric Measures

Table 8 displays the findings of the stability analysis for non-parametric measurements. The genotypes G8, G7, G2, G9, and G5 had the least values and were shown to be the most stable genotypes based on Si^(1)^ and Si^(2)^ statistics. Genotypes G7, G10, and G6 were more stable than genotypes G3, G9, and G2, according to two non-parametric metrics, Si^(3)^ and Si^(6)^. The most stable genotypes were G8, G2, G7, and G9 according to the NPi^(1)^ statistic; however, the most stable genotypes according to the NPi^(2)^ statistic were G8, G1, G9, and G2. The genotypes G8, G9, G1, G2, and G6 were found to be the most stable when compared to the other genotypes since they had the lowest values of NPi^(3)^ and NPi^(4)^. The genotype G7, which had the largest seed yield and the lowest rank for mean yield performance across four test conditions, was generally found to be a stable genotype according to the results of all non-parametric metrics, except for NPi^(2)^ and NPi^(4)^.

### 2.6. AMMI Stability Measure

A quantitative stability measure, which is necessary to categorize and rank the genotypes, is not included in the AMMI model. Twelve stability measures from the AMMI model were compared with the mean yield for each genotype in all environments; these are shown in Table 9. Moreover, the method proposed by Farshadfar et al. [53] was used to compute the SSI for yield and stability for each of the twelve measures of stability. The ten genotypes were then ordered according to SSI for each of the twelve stability indices from the AMMI model, with the highest ranking going to the genotype with the highest yield and stability and the lowest ranking going to the genotype with the lowest yield and instability. To analyze GEI, the ASI, ASV, MASV, and MASI are all similar to one another. Genotypes G2 and G9 were the most stable, whereas G4 was the least stable, according to the results of ASI and ASV. When the first two IPCAs account for a large amount of the variance, the parameters ASI and ASV are helpful, but their combined ability to explain variation when three or more IPCAs are relevant is limited. The genotypes G8, G2, and G9 are the most stable, whereas G3 and G4 were determined to be the least stable, according to the values of MASI and MASV, which take into account all major IPCAs. While MASI and MASV measures are identical to plotting with all significant PCA axes for ranking of genotypes, Zali et al. [54] also reached a similar conclusion when comparing ASV and MASV. ASI and ASV measures are equivalent to making a biplot with the first two PCA axes. The total across environments of the absolute value of the GEI modeled by AMMI (AVAMGE) parameters is the total across environments of the absolute value of the eigenvectors. The least stable genotype was G3, which had the highest absolute value of AVAMGE; the most stable genotypes were G8, G2, and G9. DA and DZ as well as the fitted AMMI model (FA) and the AMMI-based stability parameter (ASTAB) all indicated genotypes G8, G2, and G7 as the most stable in this investigation, whereas genotype G3 was the least stable. The most stable genotypes were G8, G7, and G2, while G3 was the least stable, according to the sums of the absolute values of the IPC scores (SIPC). The G8, G2, and G7 genotypes were shown to have the highest levels of stability, whereas the G3 genotype had the lowest levels of stability, according to the absolute value of the relative contribution of IPCs to the interaction (Za).

### 2.7. Parametric Stability Measures

Table 10 lists the results of parametric stability measurements for each genotype. Table 10 displays the findings of Eberhart and Russell’s [20] investigation. The main impacts of G and E as well as the GEI were significant (*p* ≤ 0.01). Through the slope of the regression line (bi) and variance in the regression deviation, the joint regression estimated the stability of each genotype (Sdi^2^). The genotypes G6, G7, and G10 were broadly suited to all test conditions because they possessed bi ≈1, which resulted in better grain production than the average yield performance. In high-yielding conditions, genotypes G1 and G3 are appropriate due to their bi > 1 and low rates of average stability. The grain yield of genotypes G2 with bi < 1 was greater than the average yield performance, making them well adaptable to poor-yielding environments (Table 10). The genotypes G8, G5, G2, and G1 with the lowest values had the highest levels of stability according to the S^2^di statistic. In this regard, across four test locations, genotype G7 had the greatest seed yields, and genotype G8 had the lowest seed yields. Genotypes G8, G2, and G7 were found as the most stable genotypes based on Pinthus’s coefficients of determination (R^2^), stability variance (σ^2^i), and Wricke’s ecovalence (W_i_^2^). The most stable genotypes were determined using Francis and Kannenberg’s coefficient of variation (CV) method, which included genotypes G5, G7, G2, and G6. According to Tai stability (Tai) indices, the most stable genotypes were G9, G2, G10, and G8. The genotypes with the highest stability, as measured by the Lin and Binns’ superiority index (Pi) statistic, were G7, G10, G4, and G6, respectively.

### 2.8. Simultaneous Selection Indices (SSI) of Genotypes for Yield

These stable genotypes could not be high-yielders; stability alone is not a suitable selection criterion, for which the use of SSI as a single non-parametric measure is needed [53]. Therefore, phenotypic characteristics and stability must both be included in a single selection measure. By summing the rankings of the stability index/parameter and mean yields, Farshadfar’s [53] SSI, also known as the GSI or YSI, was calculated. High SSI is seen to be least stable with low yield, whereas low SSI is thought to be most stable with high yield. Table 11 lists the twelve stability metrics from the AMMI model that reflect the SSI rank orders. Genotypes G7, G10, and G2 were shown to be the most stable and high yielders in the current investigation, while genotype G3 is the least stable and produces poor yields, according to the SSI computed using all stability metrics.

### 2.9. Best Linear Unbiased Prediction-Based Stability Indices (BLUP)

The BLUP values, such as HMGV, RPGV, and HMRPGV (Table 12), were evaluated using BLUP-derived values for grain yield to determine which technique is best for choosing stable and high-yielding genotypes [52]. According to the stability metrics, such as HMGV, RPGV, and HMRPGV, the genotypes G7, G10, and G4 were identified as being very stable and high-yielding genotypes, whereas the G1 genotype exhibited the least stability throughout the investigated conditions.

### 2.10. Correlation between Stability Statistics

**Figure 3:**
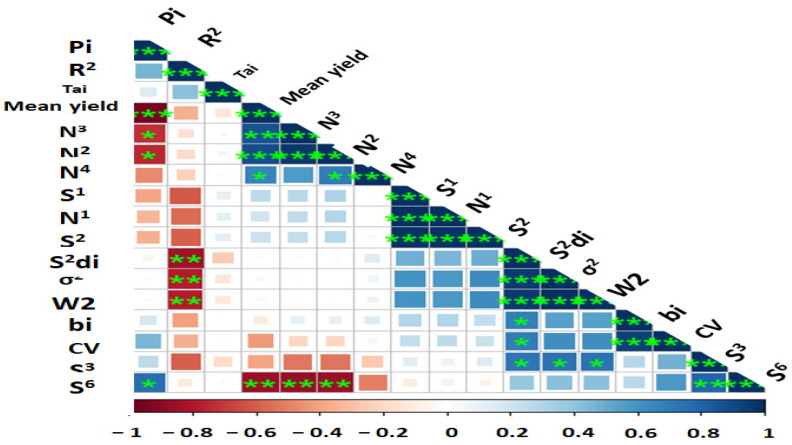
Wi^2^—Wricke’s (1962) ecovalence; b_i_—regression coefficient; S^2^di—deviation from regression; σ^2^—Shukla’s (1972) stability variance; CV—Francis and Kannenberg’s (1978) coefficient of variance; Tai—Tai stability; R^2^—Pinthus’s (1973) coefficients of determination; S^(i)^—Huehn’s (1979) and Nassar and Huehn’s (1987); NP^(i)^—Thennarasu’s non-parametric (1995) measures *, ** and *** significant at 0.05, 0.01 and 0.001 probability levels, respectively.

In an attempt to reveal the relationship between each pair of AMMI stability parameters, Pearson rank correlations (Figure 2 and Supplementary Table S2) revealed a strong association among the estimated AMMI-based indices except AMGE and AVGE. BLUP-based stability parameters had non-significant correlations with AMMI stability indices, but RPGV, HMRPGV, and HMGV were highly significant positive correlations with each other. According to the results, ASI showed no correlation with most of the parameters, while MASV was associated with other parameters. A heatmap-based Pearson correlation coefficient was used to investigate relationships between mean grain yield and the calculated stability indices (Figure 3 and Table S1). Based on the results, mean yield correlated positively and significantly with Pi, NP^(2)^, NP^(3)^, NP^(4),^ and S^(6)^. Other positive and significant associations were observed between the following stability statistics: Pi with NP^(2)^, NP^(3)^, and S ^(6)^; R^2^ with S^2^di, σ^2,^ and W^2^; NP^(3)^ with NP^(2)^ and NP^(4)^ and S^(6)^; N^(4)^ with S^(1)^, N^(1)^ and S^(2)^; S^(2)^ with S^2^di, σ^2^, W^2^di, bi, CV and S^(3)^; S^2^di with R^2^ (Figure 3 and Table S1).

### 2.11. Polygon View of GGE Biplot (Which-Won-Where Pattern)

**Figure 4:**
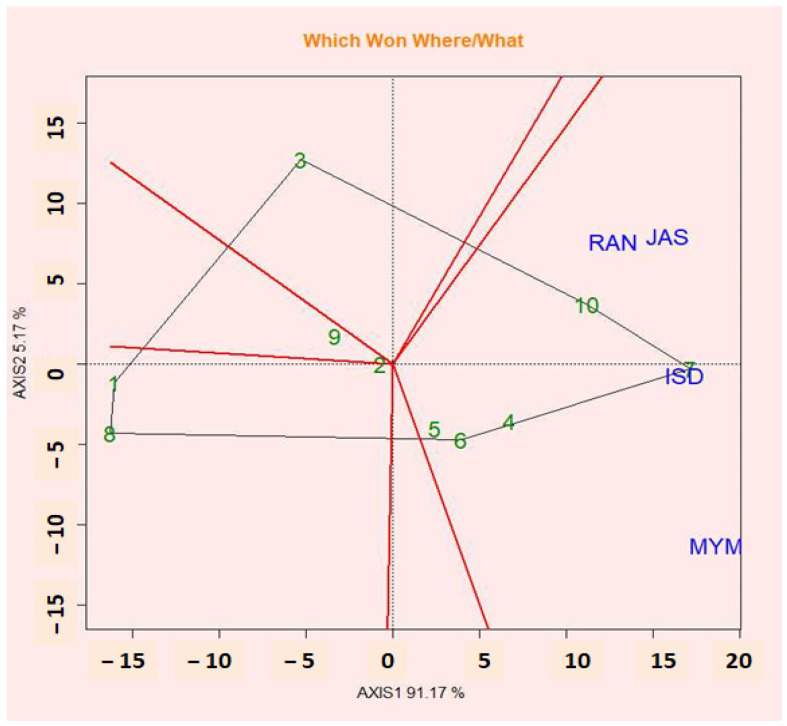
The polygon (which-won-where) shows the (G + G × E) interaction effect of 10 lentil genotypes.

The symmetrical singular value partitioning method was used to display the biplot of PC1 against PC2 for both genotypes and environments featuring the which-won-where perspective on the GGE biplot of multi-location trial data. This method is useful for understanding how genotypes and environments interact (Figure 4). An effective technique for visualizing the patterns of genotype and environment interaction is the “which-won-where” pattern [55]. Based on the GGE biplot polygonal viewgraph, the environmental adaptation of genotypes was evaluated (which-won-where graph). The GGE biplot genotype graph was used to pick the ideal genotypes for their characteristics, and the GGE biplot polygon graph was used to identify the genotypes that were best suited for the specific season. The winning genotype for an environment or set of environments during a sector is the vertex genotype. In all test environments, the vertex genotype in a sector with no environment is thought to have performed poorly. It displays the presence or lack of G × E cross-interaction, which explains how many mega-environments could occur [56]. Compared to the vertex genotypes, the genotypes inside the polygon responded less to the location. The polygon in the polygonal view (which won where) (Figure 4) of the GGE biplot is divided into a sector by perpendicular lines (rays) passing from the sides. The GGE biplot is a useful tool for visualizing GEI vs. ME genotypes and exhibiting which-won-where patterns and stable genotypes with greater performance. The results of the GGE biplot showed that the richest and poorest points, which were situated at the top of the polygon and either positively or negatively responded to the seed yield, were G7, G10, G3, G1, G8, and G9. G10 had the highest wins in JAS, RAN, and ISD, while G7 had the best winnings in ISD. G6 also did well in MYM.

### 2.12. Mean vs. Stability Views of GGE Biplot

**Figure 5:**
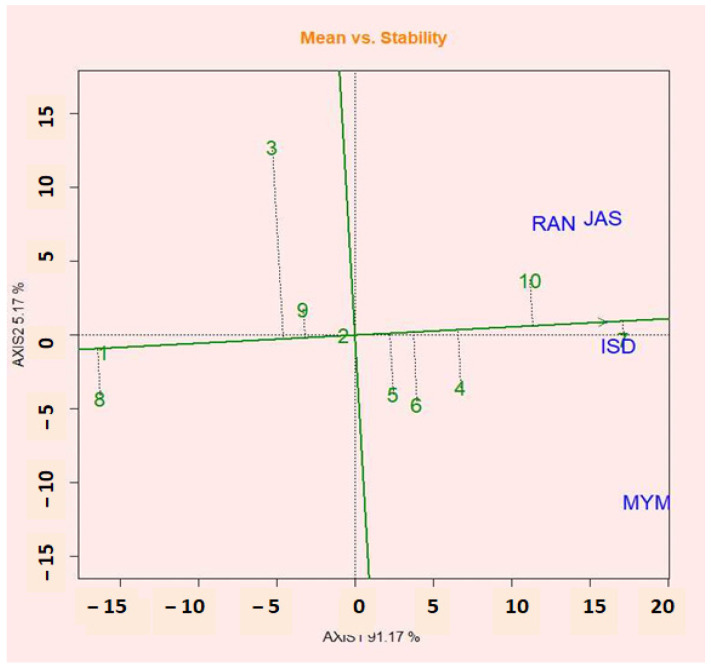
The mean vs. stability view shows the genotype main effects plus the genotypic environment interaction effect (G + G × E) of 10 lentil genotypes.

Figure 5 shows the GGE biplot analysis graphic of the 10 potential lentils in four environments. The line crossing the biplot’s origin based on sections of singular values (SVP = 1) is the mean coordinate of the environment (AEC). According to Yan et al. [36], the first principal component (PC1) of the graphic analysis reflected crop productivity, while the second principal component (PCA2) was related to genotypic stability or instability. Yan and Tinker [57] presented an ideal genotype with a high mean and relatively steady performance under the contract for explicit selection. The projection of the AEC abscissa, therefore, examines the stability of the genotypes. The genotype with the shortest contract in any direction with the AEC abscissa is thought to be unstable, whereas the genotype with the longest contract in any direction with the AEC abscissa is seen to be more stable. According to the shorter AEC axis in the figure, genotypes G7, G10, G4, and G5 are more stable and had high mean yields, whereas the G2 genotype was the most stable but had a lower yield than the mean yield. Researchers believe that the stable genotypes G7, G2, and G10 have good performance and stability at the same time.

### 2.13. Evaluation of Genotypes Relative to Ideal Genotypes

**Figure 6:**
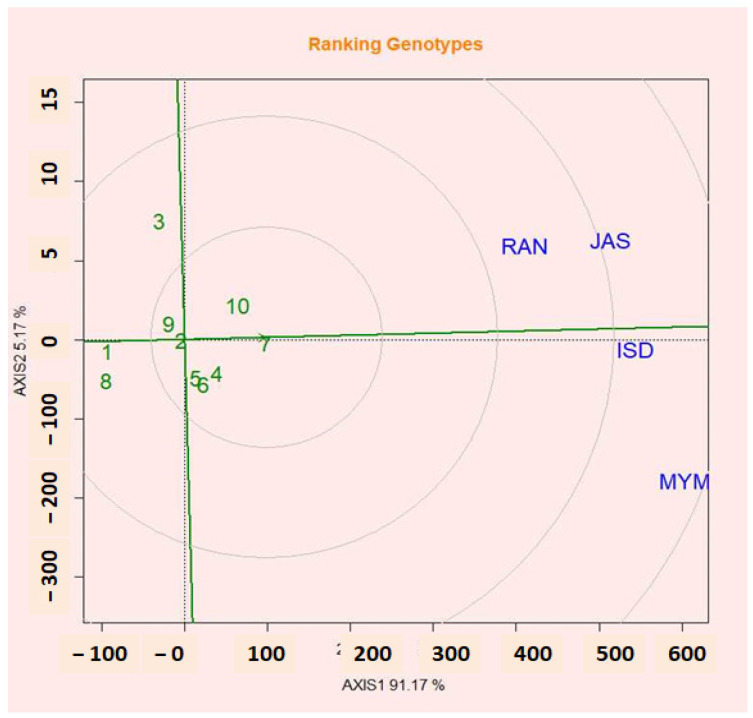
GGE biplot showing the ideal genotype and comparison of the genotypes with the ideal genotype.

The categorization of lentil genotypes according to the best genotype for seed yield is shown in Figure 6. A genotype is said to be ideal if it consistently produces the highest yield across all environments [57]. High-producing and more stable genotypes may be ideal [58]. Absolute stability was demonstrated at the smallest distance from the optimum genotype. The genotypes located near the concentric circle an almost ideal genotype were also highly productive and stable [59,60]. Figure 6 shows that G7, which performed the best after G10, G4, and G5, had high means and stability. The ideal variety approach of the GGE biplot was used in this work to determine the relative contributions of stability and seed yield to the identification of good varieties [49].

### 2.14. Evaluation of Environments Relative to Ideal Environments

**Figure 7:**
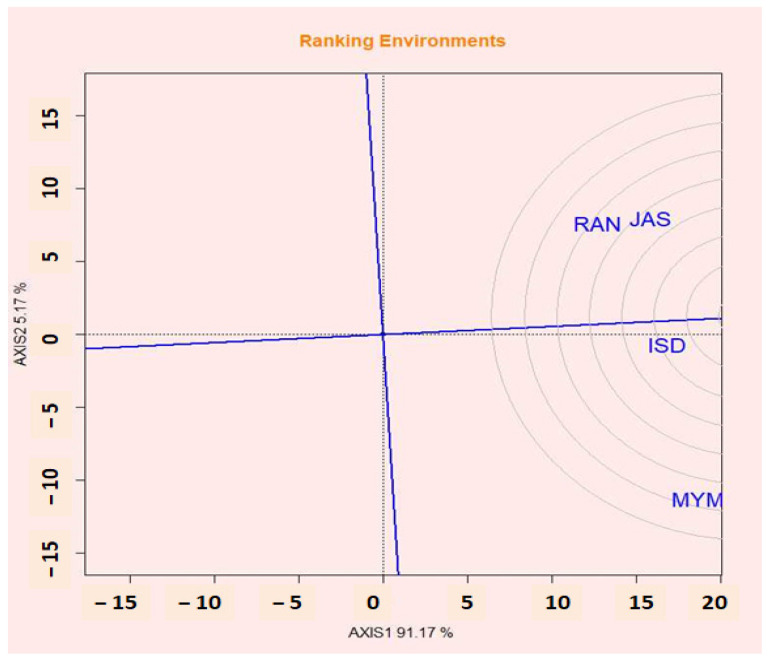
GGE biplot shows the performance of environments relative to the ideal environment.

According to Yan and Kang [61], the ideal environment might be both highly discriminating for the genotypes tested and reflective of the intended environment. Similar to the ideal genotype, an ideal environment is defined and shown by a little circle with an arrow pointing toward it. The effectiveness of lentil genotypes changed significantly in each of the four tested locations (Figure 7). Assuming that this is the case, a perfect environment is represented by a tiny circle in the middle of several concentric circles with an arrow pointing at it. ISD was chosen as the most desired test environment because it was the most representative of the general environment and powerful in discriminating lentil lines.

### 2.15. Discrimination Abilities vs. Representation of Environments

**Figure 8:**
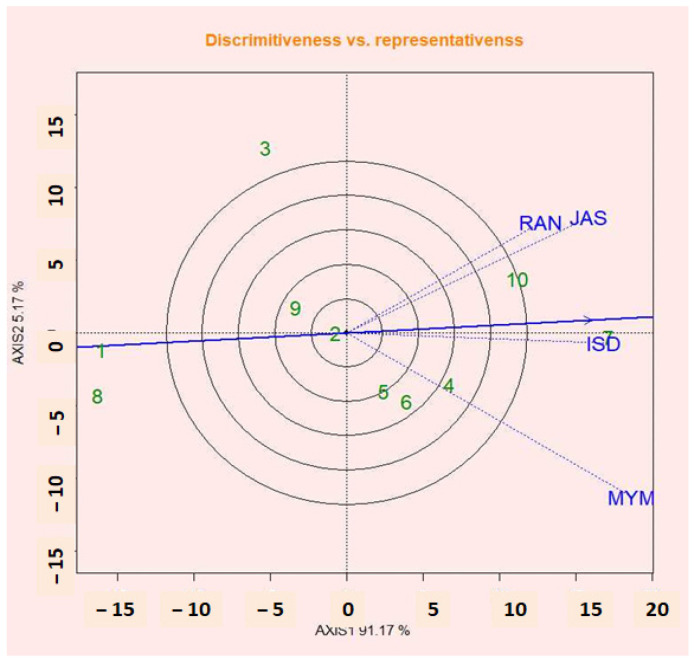
Discrimination ability vs. representativeness for four testing environments.

**Figure 9:**
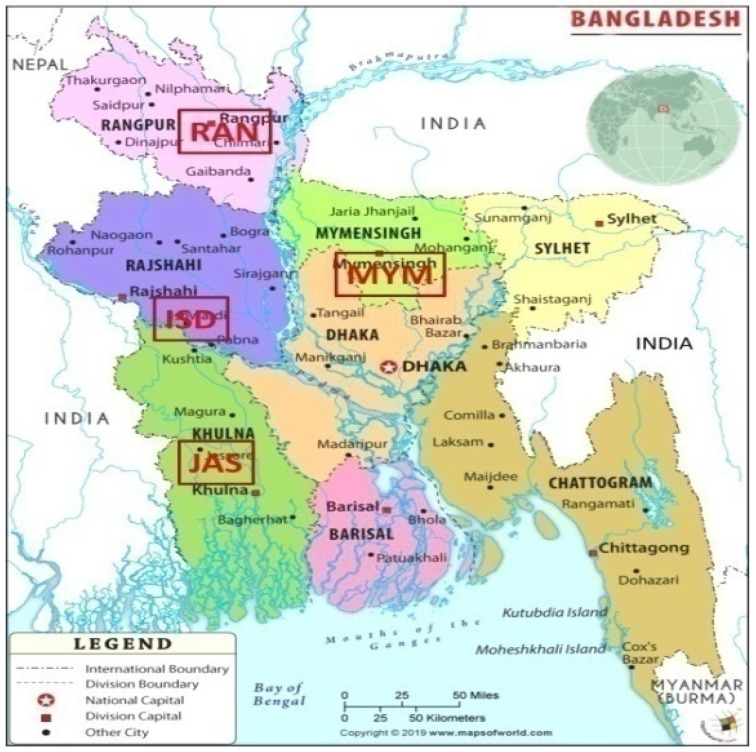
Study sites showing on the map. JAS = Jashore; ISD = Ishwardi and MYM = Mymensingh.

The most crucial steps in test environments that give not only valuable but unbiased information about the tested genotypes are the discriminating ability and representativeness viewpoint of the GGE biplot [37]. A critical measurement in the GGE biplot is the testing environments’ discriminating power and representativeness. The concentric circles in Figure 8 assist to see the length of the environment vectors, which is calculated by the discriminating ability of the environments and also as variance within the respective environments [57]. Longer vector environments are highly discriminative to all or any tested genotypes [61]. The findings of this study were consistent with those of Fayun et al. [61], who noted that genotype variation was low for short vectors and high for strongly projected test locations. The capacity of test sites to distinguish between lentil genotypes as shown in Figure 8 is in the following order: MYM, JAS, ISD, and RAN. According to the theory that the obtuse angle between the location axes and the AEC axis showed a negative correlation [62], MYM is not appropriate for the location of the lentil (Figure 9). ISD is the most representative and discriminating test site for lentil selection. The strongest projections of G1, G6, and G9’s means from the center of the biplot were the best. The richest genotypes that were positively deviated are G7, G10, and G2 (Figure 8). This resulted in the genotypes for mean performance being inconsistent as a result of a significantly substantial GEI impact in multi-environmental trials (METs).

## 3. Discussions

Lentils play an essential role in the food and nutritional safety of millions, especially in Asia; therefore, even a slight increase in their nutritive content may be extremely significant for the improvement of human nutrition. The varied weather conditions were noticed, during the four separate sites, where the experimental substance was studied. The recorded precipitation and temperatures were found to be varied at every location and consequently influenced the seed production of the examined genotypes.

In our study, ANOVA has shown a noteworthy difference among traits. The literature has also shown corroborative results in agronomic traits of mungbean [63], *Zea mays* [64,65,66,67], *Oryza sativa* [68,69,70,71,72,73,74,75,76,77,78,79,80,81,82,83,84], okra [85,86,87], *A.* spp. [88,89], broccoli [90], and *Cocos nucifera* [91,92]. In contrast to the environment and genotype–environment interactions, AMMI analysis of variance revealed that genotype was the largest contributor to the overall variation in seed yield. Grain yield exhibited significant differences between environments, genotypes, and GEI at 0.1% (*p* ˂ 0.001), according to mean squares from the combined ANOVA. For every characteristic that was reported, the AMMI analysis found significant variance (*p* < 0.001) among the genotypes, environments, and GEI. According to yield, the environment and GEI contributed about 22.79 and 69.14%, respectively, whereas genotypes contributed 91.99% of the total variance. Moreover, the research showed that the first two PCs strongly explained GEI. The first PC among them made up 61.3% of the total GEI, which was followed by the second and third PCs at 23.3 and 15.4%, respectively. As a result, AMMI1 (IPCA1 versus additive main effects) and AMMI2 (IPCA2 vs IPCA1) biplots were created to demonstrate the simultaneous impacts of genotype and environment. The yields in the other location were below the environmental mean, according to the AMMI1 biplot, but JAS and ISD were high-yielding environments with large additive genotypic main effects. The genotypes in this biplot’s scatter plot showed that the genotypes G10, G7, and G5 had the highest yields.

The genotypes with higher mean values, regression coefficient value less than unity, and non-significant deviations from linear regression deviation (S^2^di = 0) were considered adaptable to unfavorable environmental conditions, while the genotypes with higher mean values, regression coefficient value more than unity, and non-significant deviations from linear regression deviation (S^2^di = 0) were considered adaptable to favorable environmental conditions, and genotypes with higher mean values, regression coefficient value near unity and non-significant deviations from linear regression deviation (S^2^di = 0) were considered adaptable to varied environmental conditions. Eberhart and Russell’s 1996 [20] model has been widely used to find out stability parameters through genotypes × environment interaction i.e., mean seed yield, regression coefficient, and deviation from regression. It revealed a significant effect of each environment on the lentil genotypes taken for all the six agro-morphological traits. It is noted that the genotypes G2, G4, G7, and G9 were stable for all the characters in all the environments.

Our research showed that the contribution of AMMI2 to the GEI sum of squares was in concurrence with the proportion of AMMI2 in the total variance in Jamshidmoghaddam and Pourdad [93] and with the findings of a study by Islam et al. [94], which demonstrated that the AMMI2 biplot may be more accurate at extracting GEI variation given that it contains data from two IPCAs. Because the AMMI2 model was easy, inferences concerning stability, genotypic performance, genetic divergence between cultivars, and the environments that improve cultivar performance could be drawn [95]. In the AMMI2 biplot graph, close genotypes and environments show positive relations and stable genotypes are located close to the biplot’s origin [96,97]. In the current study by AMMI2, some genotypes (G7, G6, and G10) were adapted to the testing environment based on yield. Even though the environmental factor was not as significant, genotype interaction and the particular environment were able to account for a significant amount of variance. However, less environmental fluctuation, such as changes in temperature and rainfall, resulted in the inconsistent performance of lentil genotypes in Bangladesh’s northwestern region. Studies by Shrestha et al. [4] found that environmental variation significantly contributed to the total variation in lentils, whereas studies by Dehghani et al. [98], Hamidou et al. [36], and Singamsetti et al. [99] revealed GEI.

Different stability statistics produced similar ranking patterns in the selection of stable genotypes, indicating that any of them would be appropriate for choosing desirable genotypes. The stability parameters, namely, S^(1)^, S^(2)^, S^(3)^, S^(6)^NP^(1)^, NP^(2)^, NP^(3)^ and NP^(4)^, ASI, ASV, ASTAB, AVAMGE, DA, DZ, EV, FA, MASI, MASV, SIPC, Za, HMGV, RPGV, and HMRPG, were computed to compare whether they were equally efficient in assessing the stability of genotypes. The genotypes G7, G10, and G6 were more stable than genotypes G3, G9, and G2, according to two non-parametric metrics approaches S^(1)^, S^(2)^, NP^(2)^, NP^(3)^ and NP^(4)^. Among the genotypes, extremely low scores and minimal variance across the genotypes were recorded for characteristics such as EV and Za, calculating that they would not be particularly useful in further SSI calculations. A genotype is more stable in any stability measure if its score is lower and vice versa. The most stable genotypes are G2 and G10, according to ASI and ASV calculations. The most stable genotypes in EV, MASI, and MASV are G8, G2, and G9. The genotypes G8, G2, and G7 were shown as the most stable genotypes depending on the bi, Sdi^2^, R^2^, σ^2^i, and W_i_^2^. However, as a whole, all the stability parameters almost showed a similar pattern in identifying stable genotypes. Similar findings were reported in the study by Shrestha et al. [4] in lentils using the same set of stability indices.

The present study’s findings revealed a significant association between mean yield (GY) and the stability statistics for Pi, S^(2)^, S^(3)^, S^(6)^, NP^(2)^, NP^(3)^, NP^(4)^, ASI, ASV, KR, bi, Za and ASV stability statistics. Because these data are connected to the dynamic concept of stability, making decisions based on each of them is acceptable [100]. The findings from Ahmadi et al. [101] are in agreement with this conclusion. The findings demonstrated a positive and significant correlation between the majority of parametric and non-parametric statistics, including S^(1)^, S^(3)^, S^(6)^, NP^(2)^, bi, W2, θi’, Sdi^2^ and R^2^ as well as all AMMI-based stability statistics (ASV, SIPC, EV, and Za). Significantly favorable relationships between the σi^2^, Wi and Pi were observed. ASV with CV and σi^2^ with Wi all had positive correlations with CV, indicating that these measures may always be used to identify stable wheat genotypes.

The performance of tested genotypes in various contexts may be compared using GGE biplots to identify differences across environments with different features. A polygon representation of the genotypes that shows “which-won-where” is one of these options. The genotype markers that are furthest away from the biplot origin in each direction are the vertices of the polygon, which contains all genotype markers. The vertex genotype for each sector is the one with the highest yield for the environments that fall within that sector. This study’s polygon plot revealed two mega-environments with distinct winning genotypes, demonstrating how a genotype may be specifically adapted to a mega-environment and how the GE interaction can be put to good use. The most particular adapted genotypes for yield under JAS, RAN, and ISD were G10 and G7. For each location and the averaged data, the first two PCs explained 93.28% of the total variability brought on by genotype (G) and genotype-by-location (GL) effects. Jamshidmoghaddam and Pourdad [93] in safflower (*Carthamus tinctorius* L.) and Vaezi et al. [30] in barley (*Hordeum vulgare*).

In this work, we identified the optimum genotype using the mean versus stability view of the GGE biplot. According to Yan and Kang’s theory, the optimum genotype should have a high mean yield as well as great environmental stability [49]. The optimal genotypes in this study were determined to genotype G7, G10, G4, and G5, which had a high mean yield and excellent stability. In the GGE biplot graphs, the environment with the shortest angle with AEA is the most representative [57]. While a non-discriminating environment that plots close to the origin of the biplot is not informative, gives little information, and should not be utilized as a test environment in plant breeding studies proposed at cultivar release, it is the most informative and discriminating environment [57,93]. While discriminating and representative environments are efficient test environments to confirm widely adapted genotypes, discriminating and non-representative environments are suitable to test environments to find genotypes with unique adaptations. The most discriminating and representative environment was ISD for seed yield, according to the environment-focused scaling GGE biplot. A test environment can be identified by its similarity to other environments and its ability to make distinctions [44]. The GGE biplot analysis’s findings agreed with earlier studies on yield, which showed that it was more responsive to environmental variables than seed yield in several crops by Vaezi et al. [30] in barley and soybean studies by Dallo et al. [102].

## 4. Materials and Methods

### 4.1. Planting Sites and Plant Materials

The study was carried out as nine improved lines and one popular variety of lentil (Table 13), which was developed by the International Organization of International International Council for Agricultural Research in the Dry Areas (ICARDA), and one released variety as a check BARI Masur-8 from the Bangladesh Agricultural Research Institute (BARI). In four test locations, these sites were chosen to represent three different agro-climatic zones (AEZs). The longitude, latitude, soil type, precipitation, and environmental factors of those test ecological areas (Table 14 and Figure 9).

### 4.2. Experimental Design and Data Collection

A randomized block design with three replications was used to arrange the treatments. Each genotype’s seeds were planted in the winter (mid-November) and collected in the last week of February. Four environments were used generally for the evaluation. Plots were made up of six rows 3 m long with a row-to-row and plant-to-plant spacing of 40 and 10 cm, respectively, in each environment. Furrowing, cross-furrowing with a state furrow, and laddering was used to complete the land layout. At the time of the final land preparation, the soil was fertilized with 20-40-20-10 N-P-K-S kg per hectare in the form of urea, triple superphosphate, muriate of potash, and gypsum, separately BARI [103]. Other agronomic procedures were applied consistently over the whole experimental region.

Five randomly selected plants from the middle rows of each plot in each environment were studied for data on their days to flowering, days to maturity, plant height in centimeters, number of pods per plant, the weight of 100 seeds, and yield per square meter, which was converted to kilograms per hectare (YPH).

### 4.3. Statistical Analysis

We averaged each treatment from all the sample data of a trait to obtain a replication mean [104,105,106,107,108,109,110]. The average data of various traits were analyzed statistically [111,112] and biometrically [113,114]. We used Statistix 8 software for obtaining an analysis of variance (ANOVA) [115,116]. Using the package “metan” in R 4.1.3 [117] (R Team, 2020)**,** the AMMI analysis was carried out [118]. Twenty-four stability statistics were calculated, and further genotypes were ranked based on each statistic (Table S3). All AMMI-based stability statistics were computed using the R “metan” package. Additionally, a web-based STABILITYSOFT application was used to calculate several parametric and non-parametric stability statistics [119]. Simultaneous selection indices (SSI) using the AMMI stability, this strategy is based on average yield and stability, as described by Rao and Prabhakaran [38]. Using the “lme4” package in R, the estimation of BLUP-based stability models such as HMGV, RPGV, and HMRPGV was carried out [120]. The mean data were analyzed graphically according to Yan and Tinker [57] using R 4.1.3 software and GGEBiplotGUI tools. There are four methods to convey the findings: (1) polygon biplot display to analyze the genotypes capacity to adapt to specific environments across testing sites (which-won-where pattern of GGE), (2) the identification of the ideal genotype(s) based on mean and stability, (3) the selection of the best testing locations, and (4) the graphical display of concentric circles with vectors of entries that reveal associations between various environmental factors and genotypes to identify stable genotypes. Using the corrplot program in R software, the correlation matrix was calculated.

The parameters of stability indices such as regression coefficient (bi), deviation from regression (S^2^di) and phenotypic index (Pi) were calculated according to methods described by Eberhart and Russel [20]. The equation is below

bi = [∑j YijIj/I2](1)

where ∑Yij Ij is the sum of the product of the environmental index (Ij) with the corresponding mean of that genotype of each environment. The phenotypic index may be used to determine a genotype’s superiority (Pi). The better genotype is the one with the little Pi value that has consistently been among the most productive in a given set of locations.

S^2^di = [∑j δ^2^ij/(e−2)−S^2^e/r] (2)

where S^2^e = estimated pooled error and r = no. of replication $\sum \delta^{2}$ ij = [$\delta^{2}$ vi − bi ∑YijIj], which is the variance of mean over different environments with regard to individual genotypes.

(3) $$\mathrm{Pi}=\overset{-}{Y}i.-\overset{-}{Y}$$

where $\overset{-}{Y}$i = mean of the ith genotype over the environment, and $\overset{-}{Y}$ = overall mean

The environmental index (Ij) is defined as the deviation of the mean of all the genotypes at a given environment from the overall mean. An environmental index can be calculated to determine which environmental factors contribute to poor, fair, or ideal growth conditions. The environmental index reflects the adequacy of an environment to exhibit a specific characteristic. The positive and negative environmental indices (Ij) indicate the character’s favorable and unfavorable environments, respectively. A positive environmental index indicates increased grain yield, whereas a negative index indicates decreased grain yield.

## 5. Conclusions

Among 10 genotypes assessed at four different sites, the GE interaction as well as the genotype and environment main effects were shown to be significant. Genotypes had a significant impact on the performance of the advanced lentil lines, which was followed by environmental factors and then the GEI. The discovery of lentil lines that explained both general and detailed adaptation to the investigated environments was made possible by significant GEI. Therefore, multi-environmental trials are useful for choosing the best genotypes that have a high yield and a high level of stability. Effective graphical techniques for analyzing GEI in lentil multi-environment yield experiments include AMMI and GGE biplot models. Moreover, combining approaches from both the numerical and the graphical techniques might help breeders in developing more accurate regional recommendations for genotype release, depending on adaption and stability. Overall, 8 out of 33 statistics also revealed a substantial positive association with yield, making all stability statistics positive or negative and highly connected with one another. According to the stability analysis graphically and Eberhart and Russel, the genotypes G7, G2, and G9 were identified as the best materials for rainfed conditions in Bangladesh using both numerical and graphical approaches. The highest-producing and most stable genotypes across all tested sites were found to be G7, G2, and G9. To be released as a variety, these genotypes can be added to the national testing program.

## Figures and Tables

**Table 1:** Performance of lentil genotypes for days to flowering in different environments.

Entries	Code	Days to Flowering					Pi	bi	S^2^di
		Ish	Jas	Mym	Ran	Mean			
LRIL 18–102	G1	63	55	57	60	59	29.7	1.50	−3.18
LRIL 21–139	G2	61	56	56	61	59	29.8	1.15	−1.67
ILL 2580	G3	66	62	60	65	63	8.96	0.92	−1.05
LRIL 22–165	G4	62	56	57	59	58	29.4	1.17	−3.18
LRIL 21–198	G5	60	51	53	53	54	69.3	1.50	0.76
LRIL 22–158	G6	61	55	53	58	57	46.8	1.34	−1.08
RL 12–171	G7	64	63	58	65	62	14.2	0.77	5.39 *
LG 198	G8	67	62	58	64	63	14.3	1.29	4.71
LRIL 22–133	G9	57	55	68	58	60	24.5	−0.67 **	44.20 **
BARI Masur-8	G10	65	59	60	61	61	13	1.03	−2.93
Mean		63	57	58	60	60			
Environmental Index (Ij)		3	−3	−2	0				
CV (%)		1.85	2.69	1.98	2.98	4.69			
LSD (0.05)		3.12	2.52	3.73	2.83	2.91			

Ish = Ishwardi; Jas = Jashore; Mym = Mymensingh; Ran = Rangpur; CV = coefficient of variation; LSD = least significant difference, Pi = phenotypic index; bi = regression coefficient; S^2^di = deviation from regression; * = *p* < 0.05; ** = *p* < 0.10.

**Table 2:** Performance of lentil genotypes for days to maturity in different environments.

Entries	Code	Days to Maturity					Pi	bi	S^2^di
		Ish	Jas	Mym	Ran	Mean			
LRIL 18–102	G1	93	86	98	88	91	53.3	1.05	28.40 *
LRIL 21–139	G2	97	94	96	96	96	16.5	0.32 *	0.14
ILL 2580	G3	100	91	96	90	94	23.8	1.70 *	2.09
LRIL 22–165	G4	96	94	89	92	93	44.2	0.53 *	8.17 *
LRIL 21–198	G5	95	91	88	89	91	57.5	0.87	6.90 *
LRIL 22–158	G6	98	94	91	94	94	28.4	0.68	5.20 *
RL 12–171	G7	106	97	91	98	98	18.3	1.45 *	29.80 *
LG 198	G8	105	94	100	94	98	4.57	1.80	1.18
LRIL 22–133	G9	98	95	99	93	96	14.2	0.75	5.78 *
BARI Masur-8	G10	102	98	103	96	100	1.61	0.86	6.10 *
Mean		99	93	95	93	95			
Environmental Index (Ij)		5	2	0	−2				
CV (%)		2.94	2.34	1.80	3.67	5.17			
LSD (0.05)		2.43	2.86	2.66	3.39	4.28			

Ish = Ishwardi; Jas = Jashore; Mym = Mymensingh; Ran = Rangpur; CV = coefficient of variation; LSD = least significant difference, Pi = phenotypic index; bi = regression coefficient; S^2^di = deviation from regression; * = *p* < 0.05.

**Table 3:** Performance of lentil genotypes for plant height in different environments.

Entries	Code	Plant Height					Pi	bi	S^2^di
		Ish	Jas	Mym	Ran	Mean			
LRIL 18–102	G1	38.0	37.0	40.0	39.0	38.5	2611	1.14	−462.65
LRIL 21–139	G2	40.0	39.0	43.0	41.0	40.8	−2489	1.20	−472.34
ILL 2580	G3	42.0	41.0	42.0	43.0	42.0	−2509	1.05	−467.12
LRIL 22–165	G4	43.0	39.0	39.0	41.0	40.5	2647	−1.18	−470.36
LRIL 21–198	G5	35.0	32.0	34.0	31.0	33.0	2866	2.07	−465.56
LRIL 22–158	G6	39.0	38.0	39.0	36.0	38.0	−2629	1.08	−476.50
RL 12–171	G7	42.0	44.0	44.0	45.0	43.8	2419	2.52 *	−466.47
LG 198	G8	44.0	41.0	40.0	42.0	41.8	2603	−1.18	−471.23
LRIL 22–133	G9	32.0	35.0	38.0	35.0	35.0	2711	1.27	−458.45
BARI Masur-8	G10	35.0	40.0	38.0	38.0	37.8	2663	1.04	−468.87
Mean		39.0	38.6	39.7	39.1	39.1			
Environmental Index (Ij)		−0.2	−0.5	0.6	0				
CV (%)		2.83	2.34	1.67	1.89	3.72			
LSD (0.05)		2.29	3.23	2.12	2.79	3.34			

Ish = Ishwardi; Jas = Jashore; Mym = Mymensingh; Ran = Rangpur; CV = coefficient of variation; LSD = least significant difference, Pi = phenotypic index; bi = regression coefficient; S^2^di = deviation from regression. * significant at 5%.

**Table 4:** Performance of lentil genotypes for pods per plant in different environments.

Entries	Code	Pods Per Plant					Pi	bi	S^2^di
		Ish	Jas	Mym	Ran	Mean			
LRIL 18–102	G1	58	64	59	66	62	1049	0.38	10.60
LRIL 21–139	G2	74	68	64	70	69	727	0.75	−0.16
ILL 2580	G3	122	106	87	107	106	113	2.63	55.20 *
LRIL 22–165	G4	81	80	74	83	79	399	0.80	−5.04
LRIL 21–198	G5	80	85	69	87	80	373	1.43	11.90
LRIL 22–158	G6	74	63	70	64	68	790	−0.14	29.90 *
RL 12–171	G7	60	59	53	62	58	1176	0.71	−5.30
LG 198	G8	69	70	63	73	69	734	0.81	−3.00
LRIL 22–133	G9	68	69	63	71	68	777	0.62	−3.96
BARI Masur-8	G10	84	86	66	88	81	337	2.01 *	3.39
Mean		77	75	67	77	74			
Environmental Index (Ij)		3	1	−7	3				
CV (%)		2.35	3.16	2.51	3.14	4.23			
LSD (0.05)		4.98	2.59	3.34	5.94	3.61			

Ish = Ishwardi; Jas = Jashore; Mym = Mymensingh; Ran = Rangpur; CV = coefficient of variation; LSD = least significant difference, Pi = phenotypic index; bi = regression coefficient; S^2^di = deviation from regression; * = *p* < 0.05.

**Table 5:** Performance of lentil genotypes for 100 seed weight in different environments.

Entries	Code	100 Seed Weight					Pi	bi	S^2^di
		Ish	Jas	Mym	Ran	Mean			
LRIL 18–102	G1	1.66	1.65	1.37	1.72	1.60	0.48	1.20	0.35
LRIL 21–139	G2	1.89	1.93	1.75	2.00	1.89	0.23	0.75	0.24
ILL 2580	G3	1.23	1.16	0.96	1.25	1.15	1.02	1.00 *	0.99
LRIL 22–165	G4	1.86	1.78	1.64	1.87	1.79	0.31	0.81	0.38
LRIL 21–198	G5	2.19	2.04	1.92	2.13	2.07	0.13	0.82	0.40
LRIL 22–158	G6	2.03	2.10	1.87	2.19	2.05	0.14	0.95	0.81
RL 12–171	G7	1.69	1.57	1.36	1.67	1.57	0.51	1.13	0.55
LG 198	G8	1.75	1.71	1.50	1.81	1.69	0.39	1.04	0.84
LRIL 22–133	G9	2.43	2.64	2.41	2.74	2.55	0.04	0.78	0.32 *
BARI Masur-8	G10	2.52	2.17	1.94	2.27	2.22	0.08	2.41 *	0.03 *
Mean		1.92	1.87	1.67	1.96	1.86			
Environmental Index (Ij)		0.06	0.01	−0.19	0.10				
CV (%)		1.15	1.12	1.21	1.34	1.29			
LSD (0.05)		0.68	0.59	0.34	0.74	0.51			

Ish = Ishwardi; Jas = Jashore; Mym = Mymensingh; Ran = Rangpur; CV = coefficient of variation; LSD = least significant difference, Pi = phenotypic index; bi = regression coefficient; S^2^di = deviation from regression; * = *p* < 0.05.

**Table 6:** Performance of lentil genotypes for seed yield in different environments.

Entries		Seed Yield					Pi	bi	S^2^di
		Ish	Jas	Mym	Ran	Mean			
LRIL 18–102	G1	1164	970	786	974	973	144,789	1.38	1885.0 *
LRIL 21–139	G2	1320	1225	1108	1239	1223	41,953	0.76	1036.0*
ILL 2580	G3	1275	1253	858	1251	1159	71,198	1.51	169.0
LRIL 22–165	G4	1550	1329	1238	1227	1336	15,345	1.24	6286.0 *
LRIL 21–198	G5	1335	1259	1231	1255	1270	29,767	0.39	233.3 *
LRIL 22–158	G6	1460	1269	1220	1218	1292	23,352	0.93	4202.0 *
RL 12–171	G7	1658	1515	1425	1430	1507	5356	0.93	2291.0 *
LG 198	G8	1065	999	861	942	967	146,946	0.80	−346.3
LRIL 22–133	G9	1255	1270	1044	1148	1179	55,907	0.87	3049.0 *
BARI Masur-8	G10	1550	1515	1266	1314	1411	6304	1.21	4245.0 *
Mean		1363	1260	1104	1200	1232			
Environmental Index (Ij)		131	28	−128	−32				
CV (%)		5.45	7.52	6.71	8.84	6.89			
LSD (0.05)		45.56	48.66	52.43	49.87	38.98			

Ish = Ishwardi; Jas = Jashore; Mym = Mymensingh; Ran = Rangpur; CV = coefficient of variation; LSD = least significant difference, Pi = phenotypic index; bi = regression coefficient; S2di = deviation from regression; * = *p* < 0.05.

**Table 7:** ANOVA for AMMI model applied to yield and yield-related traits values of lentil genotypes tested in four environments.

Source	df	DF		DM		PH (cm)		PPP		Yield (kg/ ha)		Cumulative %
		MS	%Total SS	MS	%Total SS	MS	%Total SS	MS	%Total SS	MS	%Total SS	
Environments	2	256.07 **	30.69	237.21	24.58 **	2207.77 ns	7.14	948.68 **	10.50	491,049.30 **	24.06	24.06
Genotypes	9	64.67 **	34.88	92.96	43.34 **	2428.43 ns	35.33	1586.49 **	78.98	319,398.80 **	70.41	94.47
G × E Interaction	18	31.92 **	34.44	34.41	32.08 **	1976.93 ns	57.53	105.71 **	10.53	12,555.00 **	5.53	100
IPCA1	10	49.79 **	86.63	49.25	79.52 **	3550.62 ns	99.78	123.29 **	64.79	15,370.29 **	68.01	68.01
IPCA2	8	9.60 ns	13.37	15.86	20.48 **	9.81 ns	0.22	83.74 **	35.21	9035.90 **	31.99	100
Residuals	60	13.87778	-	2.96	-	1880.51	-	27.61	-	2152.37	-	

Ns, non-significant, ** significant at 1%, probability level. SS, sum of the square, df, degrees of freedom, SS (%), explained SS (%).

**Table 8:** Mean yield and non-parametric measures of stability along with their ranks for 10 lentil genotypes tested in four locations.

Genotype	Mean Yield	S^(1)^	S^(2)^	S⁽³⁾	S⁽⁶⁾	N^(1)^	N^(2)^	N^(3)^	N^(4)^
G1	973	4.17	10.92	0.50	1.00	2.75	0.29	0.30	0.11
G2	1223	3.00	6.33	1.00	0.71	1.50	0.25	0.35	0.11
G3	1159	5.17	16.92	2.92	1.23	3.25	0.46	0.53	0.22
G4	1336	5.17	16.25	0.86	0.57	3.25	1.08	0.93	0.27
G5	1270	5.33	18.00	0.70	0.50	3.50	0.78	0.82	0.11
G6	1292	4.67	13.67	0.50	0.50	3.00	0.60	0.61	0.16
G7	1507	2.67	4.67	0.07	0.14	1.50	1.50	1.50	0.67
G8	967	1.50	1.58	1.00	1.00	0.75	0.08	0.12	0.00
G9	1179	3.17	6.25	1.86	1.00	1.75	0.23	0.32	0.02
G10	1411	4.50	12.92	0.07	0.14	2.75	1.38	1.78	0.10
**Rank**									
**Genotype**	**Mean Yield**	**S^(1)^**	**S^(2)^**	**S⁽³⁾**	**S⁽⁶⁾**	**N^(1)^**	**N^(2)^**	**N^(3)^**	**N^(4)^**
G1	9	5	5	3	7	5	4	2	4
G2	6	3	4	7	6	2	3	4	5
G3	8	8	9	10	10	8	5	5	8
G4	3	9	8	6	5	9	8	8	9
G5	5	10	10	5	3	10	7	7	6
G6	4	7	7	4	4	7	6	6	7
G7	1	2	2	1	1	3	10	9	10
G8	10	1	1	8	7	1	1	1	1
G9	7	4	3	9	7	4	2	3	2
G10	2	6	6	2	2	6	9	10	3

S^i^: Nassar and Huehn’s stability parameters; NP^i^: Thennarasu’s stability parameters.

**Table 9:** Mean yield and AMMI stability measures of stability along with their ranks for ten lentil genotypes tested in four locations.

Genotype	Mean Yield	ASV	AMGE	ASI	ASTAB	AVAMGE	DA	DZ	FA	MASI	MASV	SIPC	Za
G1	973	8.16	9.95	1.90	53.73	182.30	95.01	0.58	9027.49	2.05	10.95	12.26	0.24
G2	1223	3.07	1.78	1.03	26.95	121.37	65.65	0.41	4310.49	1.14	7.10	7.94	0.14
G3	1159	4.41	3.20	8.18	180.66	377.03	222.66	0.81	49,578.69	8.18	35.17	15.73	0.53
G4	1336	35.10	6.04	3.58	69.62	224.30	121.78	0.59	14,830.81	3.58	17.01	12.29	0.32
G5	1270	15.40	−2.84	2.88	75.30	212.03	119.05	0.64	14,172.99	2.90	15.21	13.18	0.30
G6	1292	12.40	3.55	3.25	35.42	185.30	93.94	0.39	8824.79	3.27	14.27	9.13	0.25
G7	1507	14.00	3.55	2.57	18.53	131.97	70.70	0.26	4998.25	2.57	11.09	5.50	0.18
G8	967	11.00	−3.42	0.74	9.60	65.88	40.47	0.24	1637.61	0.75	4.70	4.09	0.08
G9	1179	3.18	−8.53	1.50	43.37	125.37	83.40	0.53	6955.27	1.71	9.12	10.68	0.21
G10	1411	6.43	−6.75	0.72	72.19	165.63	101.50	0.71	10,302.25	1.42	9.18	11.13	0.16
**Rank**													
**Genotype**	**ryield**	**rASV**	**rAMGE**	**rASI**	**rASTAB**	**rAVAMGE**	**rDA**	**rDZ**	**rFA**	**rMASI**	**rMASV**	**rSIPC**	**rZa**
G1	9	5	10	5	6	6	6	6	6	5	5	7	6
G2	6	1	5	3	3	2	2	4	2	2	2	3	2
G3	8	3	7	10	10	10	10	10	10	10	10	10	10
G4	3	10	9	9	7	9	9	7	9	9	9	8	9
G5	5	9	4	7	9	8	8	8	8	7	8	9	8
G6	4	7	8	8	4	7	5	3	5	8	7	4	7
G7	1	8	6	6	2	4	3	2	3	6	6	2	4
G8	10	6	3	2	1	1	1	1	1	1	1	1	1
G9	7	2	1	4	5	3	4	5	4	4	3	5	5
G10	2	4	2	1	8	5	7	9	7	3	4	6	3

SV–AMMI Stability Value; AMGE—Sum across environments of GEI modeled by AMMI; ASI—AMMI Stability Index; ASTAB—AMMI-based stability parameter; AVAMGE—Sum across environments of the absolute value of genotype × environment interaction model by AMMI; DA—Annicchiarico’s D parameter; DZ—Zhang’s D parameter or AMMI statistic coefficient; FA—stability measure based on fitted AMMI model; MASI—Modified AMMI Stability Index and MASV—Modified AMMI Stability Value.

**Table 10:** Mean yield and various parametric measures of stability along with their ranks for ten lentil genotypes tested in four locations.

Genotype	Mean Yield	CV	bi	S^2^di	Tai	W_i_^2^	Pi	R^2^	σ^2^
G1	973	14.37	1.38	2004.06	0.38	9027.49	144,789	0.94	3184
G2	1223	6.62	0.76	1155.24	0.21	4310.49	41,953	0.90	1219
G3	1159	15.71	1.51	20,252.21	−0.24	49,578.69	71,198	0.67	20081
G4	1336	10.25	1.24	6405.73	0.51	14,830.81	15,345	0.81	5602
G5	1270	3.40	0.39	395.07	0.24	14,172.99	29,767	0.87	5328
G6	1292	8.12	0.93	4321.67	−0.61	8824.79	23,352	0.78	3100
G7	1507	6.61	0.93	2410.11	−0.07	4998.25	5536.	0.86	1506
G8	967	8.35	0.80	73.23	−0.07	1637.61	146,946	0.99	105
G9	1179	8.22	0.87	3168.33	−0.21	6955.27	55,907	0.81	2321
G10	1411	9.15	1.21	4364.56	−0.13	10,302.25	6304	0.86	3716
**Genotype**	**r yield**	**CV**	**bi**	**S^2^di**	**Tai**	**W^2^**	**Pi**	**R^2^**	**σ^2^i**
G1	9	9	2	4	5	6	9	2	6
G2	6	3	9	3	2	2	6	3	2
G3	8	10	1	10	8	10	8	10	10
G4	3	8	3	9	9	9	3	7	9
G5	5	1	10	2	6	8	5	4	8
G6	4	4	6	7	7	5	4	9	5
G7	1	2	5	5	10	3	1	5	3
G8	10	6	8	1	4	1	10	1	1
G9	7	5	7	6	1	4	7	8	4
G10	2	7	4	8	3	7	2	6	7

SIPC—Sums of the absolute value of the IPC scores; Za—Absolute value of the relative contribution of IPCs to the interaction; CVi —Coefficient of variance; bi—Regression coefficient; S^2^di—Deviation from regression; Tai—Tai stability; W_i_^2^—Wricke’s stability parameter; Pi—Lin and Binns’ superiority index; R^2^i—Coefficient of determination; σ^2^i—Shukla’s stability variance.

**Table 11:** Ranking of genotypes based on simultaneous selection index (SSI) considering stability and yield.

Gen.	ASV	AMGE	ASI	ASTAB	AVAMGE	DA	DZ	FA	MASI	MASV	SIPC	Za
G1	14	19	14	15	15	15	15	15	14	14	16	15
G2	9	11	9	9	8	8	10	8	8	8	9	8
G3	18	15	18	18	18	18	18	18	18	18	18	18
G4	12	12	12	10	12	12	10	12	12	12	11	12
G5	12	9	12	14	13	13	13	13	12	13	14	13
G6	12	12	12	8	11	9	7	9	12	11	8	11
G7	7	7	7	3	5	4	3	4	7	7	3	5
G8	12	13	12	11	11	11	11	11	11	11	11	11
G9	11	8	11	12	10	11	12	11	11	10	12	12
G10	3	4	3	10	7	9	11	9	5	6	8	5

Gen.—Genotype; ASV—AMMI Stability Value; AMGE—Sum across environments of GEI modeled by AMMI; ASI—AMMI Stability Index; ASTAB—AMMI-based stability parameter; AVAMGE—Sum across environments of the absolute value of genotype × environment interaction model by AMMI; DA—Annicchiarico’s D parameter; DZ—Zhang’s D parameter or AMMI statistic coefficient; FA—Stability measure based on fitted AMMI model; MASI—Modified AMMI Stability Index and MASV—Modified AMMI Stability Value; SIPC—Sums of the absolute value of the IPC scores; Za—Absolute value of the relative contribution of IPCs to the interaction.

**Table 12:** BLUP-based mean yield and ranking of 10 lentil genotypes evaluated in four tested environments.

Genotypes	Mean Yield		HMRPGV		HMGV		RPGV	
	Kg/ha	Rank	Score	Rank	Score	Rank	Score	Rank
G1	973	9	0.78	10	955.00	10	0.79	9
G2	1223	6	0.99	6	1218.00	6	0.99	6
G3	1159	8	0.93	8	1129.00	8	0.94	8
G4	1336	3	1.08	3	1324.00	3	1.08	3
G5	1270	5	1.03	5	1269.00	5	1.03	5
G6	1292	4	1.05	4	1285.00	4	1.05	4
G7	1507	1	1.22	1	1501.00	1	1.23	1
G8	967	10	0.79	9	961.00	9	0.79	10
G9	1179	7	0.96	7	1172.00	7	0.96	7
G10	1411	2	1.14	2	1401.00	2	1.15	2

Note: HMGV_i_: to investigate the mean yield and genotypic adaptability (de Resende 2004, 2016); RPGV: Relative performance of genotypes values; HMRPGV: Harmonic mean of RPGV.

**Table 13:** Genotype names, codes, and their sources were utilized in this experiment.

Sl. No.	Genotype Name	Genotype Code	Sources
1	LRIL 18–102	G1	ICARDA
2	LRIL 21–139	G2	ICARDA
3	ILL 2580	G3	ICARDA
4	LRIL 22–165	G4	ICARDA
5	LRIL 21–198	G5	ICARDA
6	LRIL 22–158	G6	ICARDA
7	RL 12–171	G7	ICARDA
8	LG 198	G8	ICARDA
9	LRIL 22–133	G9	ICARDA
10	BARI Masur-8 (Check variety)	G10	PRC, BARI

Source: International Council for Agricultural Research in the Dry Areas (ICARDA).

**Table 14:** Characteristics of test environments and climatic conditions during lentil growing seasons 2019–2020.

Locations	Locations Code	Geographical Position		Altitude (m.a.s.l.)	Temperature (°C)		Relative Humidity (%)		Rainfall (mm)	Soil Type
		Latitude	Longitude		Max	Min	Max	Min		
PRC, Ishurdi	ISD	24.1292° N	89.0657° E	18	30.5	11.75	94.5	64.2	27.3	Clay loam
RARS, Jashore	JAS	23.1778° N	89.1801° E	11	27.5	11.50	92.4	68.6	35.6	Loamy to clay loam
BAU, Mymenshingh	MYM	24.7851° N	90.3560° E	19	30.2	13.50	93.6	63.6	25.4	Clay loam
OFRD, Rangpur	RAN	25.7466° N	89.2516° E	34	28.2	11.30	91.5	60.6	20.4	Sandy loamy to clay loam

## Data Availability

Data recorded in the current study are available in all Tables and Figures and Supplementary Files of the manuscript.

## Supplementary Materials

The following supporting information can be downloaded at: https://www.mdpi.com/article/10.3390/plants12112079/s1.
