# Supplementary material for: Integrating BLUP, AMMI, and GGE Models to Explore GE Interactions for Adaptability and Stability of Winter Lentils (Lens culinaris Medik.)
Source: Plants (Basel). 2023 May 23;12(11):2079. doi: 10.3390/plants12112079 (PMC10255267; doi:10.3390/plants12112079)
Supplement: Supplementary file 1 [file plants-12-02079-s001.zip › plants-2393008-supplementary.pdf]

Table S1. Correlation coefficient between parametric and nonparametric stability measures for seed yield of lentil genotypes.

	Mean yield	CV	bi	S^2di	Tai	W^2	Pi	R^2	σ^2	$S^{(1)}$	$S^{(2)}$	$S^{(3)}$	$S^{(6)}$	$N^{(1)}$	$N^{(2)}$	$N^{(3)}$	$N^{(4)}$
Mean yield	1																
CV	-0.42	1															
bi	-0.11	0.93	1.00														
S^2di	0.02	0.70	0.68	1.00													
Tai	-0.13	0.01	0.01	-0.26	1.00												
W^2	-0.04	0.61	0.54	0.95	-0.13	1.00											
Pi	-0.95	0.46	0.18	-0.07	0.15	-0.02	1.00										
R2	-0.35	-0.35	-0.40	-0.84	0.42	-0.78	0.47	1.00									
X2	-0.04	0.61	0.54	0.95	-0.13	1.00	-0.02	-0.78	1.00								
S(1)	0.26	0.24	0.29	0.48	0.12	0.59	-0.40	-0.62	0.59	1.00							
S(2)	0.22	0.22	0.25	0.50	0.12	0.63	-0.36	-0.60	0.63	0.99	1.00						
S(3)	-0.40	0.48	0.28	0.73	-0.19	0.73	0.26	-0.59	0.73	0.13	0.16	1.00					
S(6)	-0.85	0.58	0.27	0.39	0.02	0.41	0.76	-0.11	0.41	-0.10	-0.07	0.80	1.00				
N(1)	0.21	0.25	0.29	0.45	0.12	0.57	-0.33	-0.57	0.57	0.99	0.99	0.09	-0.09	1.00			
N(2)	0.89	-0.22	0.10	0.03	0.05	-0.01	-0.75	-0.19	-0.01	0.30	0.28	-0.53	-0.86	0.29	1.00		
N(3)	0.86	-0.22	0.10	0.02	-0.04	-0.01	-0.73	-0.16	-0.01	0.27	0.24	-0.52	-0.86	0.25	0.97	1.00	
N(4)	0.67	-0.06	0.14	0.15	0.02	0.08	-0.47	-0.23	0.08	0.00	-0.03	-0.26	-0.50	0.00	0.70	0.57	1.00

Wi^2 - Wricke's (1962) ecovalence; b_i - regression coefficient; S^2di - deviation from regression; σ^2 - Shukla's (1972) stability variance; CV - Francis and Kannenberg's (1978) coefficient of variance; Tai - Tai stability; R^2 - Pinthus's (1973) coefficients of determination; $S^{(i)}$ - Huehn's (1979) and Nassar and Huehn's (1987); $NP^{(i)}$ - Thennarasu's non-parametric (1995) measures * and **Significant at 0.05 and 0.01 probability levels, respectively

Table S2. Correlation values among various stability of 10 lentil genotypes with yield at four test environments

	Mean.yield	ACV	ASI	ASV	AMGE	ASI.1	ASTAB	AVAMGE	DA	DZ	EV	FA	MASI	MASV	SIPC	Za	HMRPGV	HMGV	RPGV
**Mean.yield**	1																		
**ACV**	-0.01	1																	
**ASI**	0.30	0.01	1																
**ASV**	0.30	0.01	1.00	1															
**AMGE**	-0.11	0.41	0.36	0.36	1														
**ASI.1**	0.04	0.60	0.11	0.11	0.38	1													
**ASTAB**	-0.02	0.66	-0.07	-0.07	0.11	0.85	1												
**AVAMGE**	0.07	0.65	0.10	0.10	0.35	0.93	0.95	1											
**DA**	0.05	0.66	0.03	0.03	0.22	0.91	0.98	0.99	1										
**DZ**	0.02	0.53	-0.06	-0.06	-0.04	0.52	0.86	0.78	0.82	1									
**EV**	0.02	0.57	-0.07	-0.07	-0.05	0.58	0.91	0.82	0.86	0.99	1								
**FA**	-0.04	0.66	-0.07	-0.07	0.19	0.93	0.97	0.95	0.97	0.73	0.80	1							
**MASI**	0.07	0.64	0.08	0.08	0.34	1.00	0.88	0.95	0.94	0.58	0.64	0.95	1						
**MASV**	0.06	0.64	0.08	0.08	0.31	0.99	0.92	0.97	0.97	0.65	0.71	0.97	0.99	1					
**SIPC**	-0.03	0.50	0.05	0.05	0.15	0.63	0.85	0.85	0.85	0.95	0.91	0.74	0.66	0.73	1				
**Za**	0.02	0.59	0.14	0.14	0.34	0.95	0.91	0.98	0.97	0.72	0.74	0.93	0.96	0.97	0.83	1			
**HMRPGV**	1.00	-0.04	0.30	0.30	-0.13	0.02	-0.05	0.04	0.03	-0.01	-0.01	-0.07	0.05	0.03	-0.06	0.00	1		
**HMGV**	1.00	-0.06	0.30	0.31	-0.12	0.01	-0.06	0.03	0.02	-0.01	-0.01	-0.08	0.03	0.02	-0.06	-0.01	1.00	1	
**RPGV**	1.00	-0.02	0.29	0.29	-0.12	0.03	-0.03	0.06	0.04	0.00	0.01	-0.05	0.06	0.05	-0.05	0.01	1.00	1.00	1

AMGE - sum across environments of genotype × environment interaction (GEI) modeled by AMMI; ASI, AMMI Stability Index; ASV, AMMI stability value; ASTAB, AMMI-based stability parameter; AVAMGE - sum across environments of the absolute value of GEI modeled by AMMI; DA - Annicchiarico's D parameter; DZ - Zhang's D parameter; EV - averages of the squared eigenvector values; FA stability measure based on fitted AMMI model; MASI - Modified AMMI Stability Index; MASV - modified AMMI stability value; SIPC - sums of the absolute value of the IPC scores; Za - absolute value of the relative contribution of IPCs to the interaction. * and **Significant at 0.05 and 0.01 probability levels, respectively.

Table S3. Parameters, indices and the equations used for the calculation of stability statistics

Sl. No.	Stability indices		Equations	References
1	SIPC	Sums of the absolute value of the IPC scores	$\mathrm{SIPC} = \sum_{n=1}^{N'} \lvert \lambda_n^{0.5}\gamma_{in} \rvert$	Sneller *et al.*, 1997
2	AMGE	Sum across environments of GEI modelled by AMMI	$\mathrm{AMGE} = \sum_{j=i}^{E}\sum_{n=1}^{N'} \lambda_n\gamma_{in}\delta_{jn}$	Sneller *et al.*, 1997
3	EV	Averages of the squared eigenvector values	$EV = \sum_{n=l}^{N'} \frac{\gamma_{in}^2}{N^l}$	Zobel, 1994
4	ASV	AMMI stability value	$\mathrm{ASV}=\sqrt{\left(\frac{\mathrm{SSIP_1}}{\mathrm{SSIP_2}} \times \mathrm{PC_1}\right)^2 + (\mathrm{PC_2})^2}$	Purchase 1997
5	DA	Annicchiarico's D parameter	$\mathrm{D_a} = \sum_{n=1}^{N'} (\lambda_n\gamma_{in})^2$	Annicchiarico, 1997
6	ASTAB	AMMI-based stability parameter	$\mathrm{ASTAB} = \sum_{n=1}^{N'} \lambda_n\gamma_{in}^2$	Rao and Prabhakaran, 2005
7	AVAMGE	Sum across environments of absolute value of genotype × environment interaction model by AMMI	$\mathrm{AVAMGE} = \sum_{j=i}^{E}\sum_{n=1}^{N'} \lvert \lambda_n\gamma_{in}\delta_{jn} \rvert$	Zali *et al.*, 2012
8	DZ	Zhang's D parameter or AMMI statistic coefficient	$\mathrm{D_z} = \sum_{n=1}^{N'} \gamma_{in}^2$	Zhang *et al.*, 1998
9	MASI	Modified AMMI stability Index	$MASI = \sqrt{\sum_{n=1}^{N'} PC_n^2 \times \theta_n^2}$	Ajay *et al.*, 2018
10	MASV	Modified AMMI stability value	$\mathrm{A} = \sqrt{\sum_{n=1}^{N'-1} \left(\frac{\mathrm{SSIPC_n}}{\mathrm{SSIPC_{n+1}}} \times \mathrm{PC_n}\right)^2 + (\mathrm{PC_{N'}})^2}$	Zali *et al.*, 2012
11	Za	Absolute value of the relative contribution of IPCs to the interaction	$A = \sum_{i=1}^{N'} \lvert \theta_n\gamma_{in} \rvert$	Zali *et al.*, 2012
12	ASI	AMMI stability index	$\mathrm{ASI}=\sqrt{[PC_1^2 \times \theta_1^2] + [PC_2^2 \times \theta_2^2]}$	Jambhulkar *et al.*, 2014
13	FA	Stability measure based on fitted AMMI model	$\mathrm{FA} = \sum_{n=1}^{N'} \lambda_n^2\gamma_{in}^2$	Raju, 2002 and Zali *et al.*, 2012
15	S^i	Nassar and Huehn's stability parameters	$S_i^{(1)} = 2\sum_j^{n-1}\frac{\sum_j^n \lvert r_{ij}-r'_{ij} \rvert}{[N(n-1)]}$, $S_i^{(2)} = \frac{\sum_{j=1}^{n}(r_{ij}-\bar{r}_{i.})^2}{(N-1)}$,	Huehn's (1979) and Nassar and Huehn's (1987)
16	NP^i	Thennarasu's stability parameters	$NP^{(1)} = \frac{\sum_{j=1}^{n} \lvert r_{ij}^* - M_{di}^* \rvert}{N}$, $NP^{(2)} = \frac{\sum_{j=1}^{n} \lvert r_{ij}^* - M_{di}^* \rvert / M_{di}}{N}$,	Thennarasu's (1995)

			$NP^{(3)} = \sqrt{\frac{\sum\left(r_{ij}^{*}-\bar{r}_{i.}^{*}\right)^2}{\bar{r}_{i.}}},$ $NP^{(4)} = \frac{2\times\left[\sum_{j=1}^{n-1}\sum_{[j'-j+1]}^{n}\left|r_{ij}^{*}-r_{ij'}^{*}\right|/\bar{r}_{i.}\right]}{N(N-1)}$	
17	$HMGV_i$	to investigate the mean yield and genotypic adaptability	$HMGV = \frac{n}{\sum_{j=1}^{n}\left(\frac{1}{GV_{ij}}\right)},$ $\text{RPGV} = \frac{1}{n}\left[\frac{\left(\sum_{j=1}^{n} GV_{ij}\right)}{M_j}\right]$ $HMRPGV_i = \frac{n}{\sum_{j=1}^{n}\left(\frac{n}{RPGV_{ij}}\right)}$	de Resende (2004, 2016)
18	W_i^2	Wricke's stability parameter	$W_i^2 = \sum\left(X_{ij}-\bar{X}_{i.}-\bar{X}_{.j}+\bar{X}_{..}\right)$	Wricke, 1965
19	σ_i^2	Shukla's stability variance	$\sigma_i^2 = \left[\frac{p}{(p-2)(q-1)}\right]W_i^2 - \frac{\sum W_i^2}{(p-1)(p-2)(q-1)}$	Shukla 1972
20	CV_i	Coefficient of variance	$CV_i = \frac{SD}{\bar{X}} \times 100$	Francis and Kannenberg (1978)
21	R_i^2	Coefficient of determination	$R_i^2 = \frac{b_i^2\sum\left(\bar{X}_{.j}-\bar{X}_{..}\right)^2}{\sum\left(X_{ij}-\bar{X}_{i.}\right)^2}$	Pinthus 1973
22	P_i	Lin and Binns's superiority index	$P_i = \sum_{j=1}^{n}\left(y_{ij}-y_{.j}\right)^2/(2n)$	Lin & Binns 1988,
23	θi	Mean variance component	$\theta i = \frac{p}{2(p-1)(q-1)}\sum_{j=1}^{q}\left(x_{ij}-\bar{x}_{i.}-\bar{x}_{.j}+\bar{x}_{..}\right)^2 + \frac{SSGE}{2(p-2)(q-1)}$ $SSGE = \sum\sum\left(x_{ij}-\bar{x}_{i.}-\bar{x}_{.j}+x_{..}\right)^2$	Plaisted and Peterson, 1959
24	$\theta_{(i)}$	GE variance component	$\theta i = \frac{-p}{2(p-1)(p-2)(q-1)}\sum_{j=1}^{q}\left(x_{ij}-\bar{x}_{i.}-\bar{x}_{.j}+\bar{x}_{..}\right)^2 + \frac{SSGE}{(p-2)(q-1)}$	Plaisted,1960
